# Frailty as a sequela of burn injury: a post hoc analysis of the “RE-ENERGIZE” multicenter randomized-controlled trial and the National Health Interview Survey

**DOI:** 10.1186/s40779-024-00568-x

**Published:** 2024-09-12

**Authors:** Adriana C. Panayi, Daren K. Heyland, Christian Stoppe, Marc G. Jeschke, Samuel Knoedler, Christian Tapking, Oliver Didzun, Valentin Haug, Amir K. Bigdeli, Ulrich Kneser, Dennis P. Orgill, Gabriel Hundeshagen

**Affiliations:** 1 Department of Hand, Plastic and Reconstructive Surgery, Burn Center, BG Trauma Center Ludwigshafen, University of Heidelberg, 67071 Ludwigshafen, Germany; 2 Clinical Evaluation Research Unit, Department of Critical Care Medicine, Queen’s University, Kingston, ON K7L 3N6 Canada; 3 Department of Anaesthesiology, Intensive Care, Emergency and Pain Medicine, University Hospital Würzburg, 97080 Würzburg, Germany; 4 Department of Cardiac Anesthesiology and Intensive Care Medicine, Charité Berlin, 10117 Berlin, Germany; 5 Hamilton Health Sciences Research, Department of Surgery, McMaster University, Hamilton, ON L8S 4L8 Canada; 6 Division of Plastic Surgery, Department of Surgery, Brigham and Women’s Hospital, Harvard Medical School, Boston, MA 02115 USA; 7 Burns Center and Plastic Surgery, Unfallkrankenhaus Berlin, 12683 Berlin, Germany

**Keywords:** Burn injury, Quality of life, Frailty, Aging, Activities of daily living

## Abstract

**Background:**

With advancements in burn treatment and intensive care leading to decreased mortality rates, a growing cohort of burn survivors is emerging. These individuals may be susceptible to frailty, characterized by reduced physiological reserve and increased vulnerability to stressors commonly associated with aging, which significantly complicates their recovery process. To date, no study has investigated burns as a potential risk factor for frailty. This study aimed to determine the short-term prevalence of frailty among burn survivors’ months after injury and compare it with that of the general population.

**Methods:**

A post hoc analysis was conducted on the Randomized Trial of Enteral Glutamine to Minimize the Effects of Burn Injury (RE-ENERGIZE) trial, an international randomized-controlled trial involving 1200 burn injury patients with partial- or full-thickness burns. Participants who did not complete the 36-Item Short Form Health Survey (SF-36) questionnaire were excluded. Data for the general population were obtained from the 2022 National Health Interview Survey (NHIS). Frailty was assessed using the FRAIL (Fatigue, Resistance, Ambulation, Illness, Loss of weight) scale. Due to lack of data on loss of weight, for the purposes of this study, malnutrition was used as the fifth variable. Illness and malnutrition were based on admission data, while fatigue, resistance, and ambulation were determined from post-discharge responses to the SF-36. The burn cohort and general population groups were matched using propensity score matching and compared in terms of frailty status. Within the burn group, patients were divided into different subgroups based on their frailty status, and the differences in their (instrumental) activities of daily living (iADL and ADL) were compared. A multivariable analysis was performed within the burn cohort to identify factors predisposing to frailty as well as compromised iADL and ADL.

**Results:**

Out of the 1200 burn patients involved in the study, 600 completed the required questionnaires [follow-up time: (5.5 ± 2.3) months] and were matched to 1200 adults from the general population in the U.S. In comparison to the general population, burn patients exhibited a significantly higher likelihood of being pre-frail (42.3% vs. 19.8%, *P* < 0.0001), or frail (13.0% vs. 1.0%, *P* < 0.0001). When focusing on specific components, burn patients were more prone to experiencing fatigue (25.8% vs. 13.5%, *P* < 0.0001), limited resistance (34.0% vs. 2.7%, *P* < 0.0001), and restricted ambulation (41.8% vs. 3.8%, *P* < 0.0001). Conversely, the incidence rate of illness was observed to be higher in the general population (1.2% vs. 2.8%, *P* = 0.03), while no significant difference was detected regarding malnutrition (2.3% vs. 2.6%, *P* = 0.75). Furthermore, in comparison with robust burn patients, it was significantly more likely for pre-frail and frail patients to disclose compromise in ADL and iADL. The frail cohort reported the most pronounced limitation.

**Conclusions:**

Our findings suggest a higher incidence of post-discharge frailty among burn survivors in the short-term following injury. Burn survivors experience compromised fatigue, resistance, and ambulation, while rates of illness and malnutrition were lower or unchanged, respectively. These results underscore the critical need for early identification of frailty after a burn injury, with timely and comprehensive involvement of a multidisciplinary team including burn and pain specialists, community physicians, physiotherapists, nutritionists, and social workers. This collaborative effort can ensure holistic care to address and mitigate frailty in this patient population.

**Supplementary Information:**

The online version contains supplementary material available at 10.1186/s40779-024-00568-x.

## Background

It is estimated that up to 33,000 people each day—7 to 12 million people per year—sustain a burn injury that requires medical care and can lead to limitations in quality of life or result in death [1]. Given the backdrop of an aging population, there has been a burgeoning interest in evaluating post-burn outcomes among older adults. The assessment of the impact of “aging” on post-burn outcomes requires analysis through the lens of frailty, whereby frailty denotes an augmented susceptibility to stressors due to decreased physiological reserve and diminished capacity to maintain homeostasis [2].

A systematic review conducted in 2023 synthesized all published research on frailty and burns, identifying 18 studies dating back to 2013, with one-third of the studies published in 2022 [3]. All studies explored frailty as a risk factor for adverse outcomes of acute burns, yet the reverse hypothesis—that burn injury itself is a risk factor for long-term frailty—remains entirely unexplored. This gap in research is significant, considering that the long-term consequences of burns align with the criteria of most frailty indices [4–11]. Despite this, a history of burns is not included in such assessments.

The hypothesis proposed here is that individuals with a history of burns may exhibit a higher prevalence of frailty compared to the general population, and these differences become apparent a few months post-discharge, which is typically when burn survivors are reintegrating into their normal lives. By highlighting the severity of this issue and outlining its impact on quality of life, we aim to identify potential opportunities and pathways for informing clinical practice, future research, and policymaking efforts.

## Methods

### Source of data for burn patients

The burn population was identified from the previously published Randomized Trial of Enteral Glutamine to Minimize the Effects of Burn Injury (RE-ENERGIZE). RE-ENERGIZE was an international, multicenter, double-blinded, randomized-controlled trial that investigated the effects of enteral glutamine supplementation (0.5 g/kg) in severe burn patients. Severe burns were defined as those of partial- or full-thickness that would necessitate surgery [12]. The data collection period for RE-ENERGIZE spanned 10 years (2011–2021), and the findings were published by Heyland et al. [12] in 2022. In summary, a total of 1209 patients from 54 burn units across 14 countries were enrolled. The eligible total body surface area (TBSA) burned criteria was: > 20% for individuals aged 18 to 39 years, > 15% within concomitant inhalation injury, > 15% for individuals aged 40 to 59 years, and > 10% for those over age 60. Therefore, patients admitted with severe burns covering an average TBSA burn of 33% underwent randomization. A total of 1200 individuals were included in the final analysis, of which 596 belonged to the glutamine group while 604 were in the placebo group. Since no beneficial effect was observed from glutamine in the original trial, both groups were combined for our burn cohort analysis. The relevant data collected encompassed details about burn centers (such as geographic regions), patient demographics [including sex, age, race/ethnicity, body mass index (BMI), substance use like alcohol or smoking], and injury specifics [such as cause and extent of burn (TBSA)], as well as outcomes [comprising length of stay in the intensive care unit (ICU), length of hospital stay (LOHS), and discharge destination].

### Source of normative data

The 2022 National Health Interview Survey (NHIS) served as the primary data source for the general population. Administered by the National Center for Health Statistics through telephone or face-to-face (household) interviews, NHIS collects annual cross-sectional data on the health status of the U.S. population. The dataset employs a multistage probability study design to ensure that the data are representative of both household and non-institutionalized civilian populations in the U.S. Additionally, there is an oversampling of Black, Asian, and Hispanic populations [13]. Eligible participants include residents living in households or non-institutional settings, including rooming houses, group homes, and homeless shelters.

### Data availability and ethical approval

This study is a post hoc analysis of the RE-ENERGIZE (NCT00985205) trial, in which the analyzed data of the burn cohort have been previously published [12]. The complete dataset is not publicly accessible due to its inclusion of sensitive information that could potentially compromise the privacy of research participants. The RE-ENERGIZE trial protocol was approved by the Research Ethics Committees at Queen’s University, Kingston, Ontario, Canada (Approval No. NCT00985205; https://clinicaltrials.gov/study/NCT00985205), and all participating centers and the informed consent form underwent review and approved by the Research Ethics Board (REB approval NO. 6013407). Before randomization, each patient or their designated surrogate provided written informed consent. All documentation regarding the ethical approval can be found in the published protocol [14]. Data concerning the normative population are openly accessible from the Centers for Disease Control and Prevention National Health Center for Health Statistics at https://www.cdc.gov/nchs/nhis/data-questionnaires-documentation.htm, 2022 NHIS document.

### FRAIL (Fatigue, Resistance, Ambulation, Illness, Loss of weight) scale

**Fig. 1:**
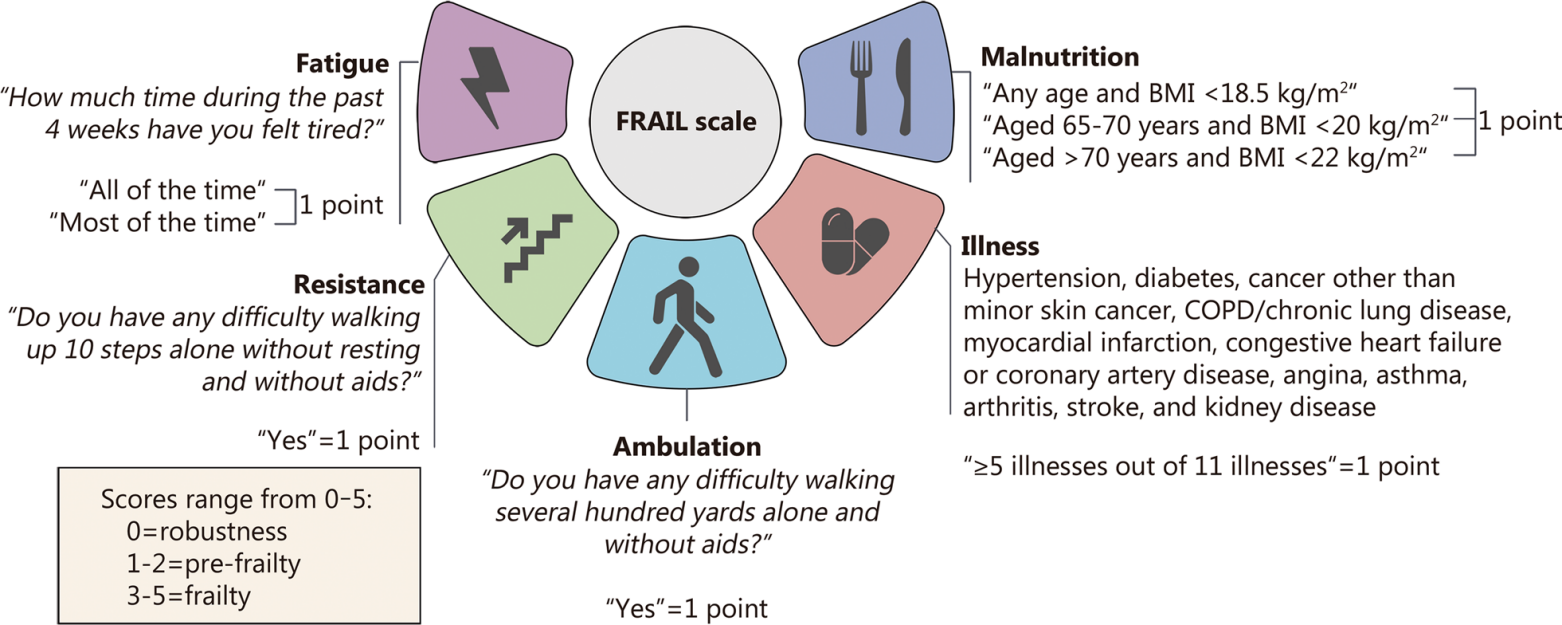
Components of the FRAIL scale. FRAIL scale is an acronym for fatigue, resistance, ambulation, illness, and loss of weight. Respondents were asked to report their level of tiredness over the past 4 weeks. Those who felt “all of the time” or “most of the time” scored 1 point on the fatigue component. Similarly, respondents were asked to query about any difficulty walking up 10 steps alone without resting or aids. Those answering “Yes” scored 1 point on the resistance component. Additionally, respondents were asked if they had any difficulty walking several hundred yards alone without aids. A positive response also scored 1 point on the ambulation component. Finally, individuals reporting 5 or more out of 11 specified illnesses (hypertension, diabetes, cancer other than minor skin cancer, COPD/chronic lung disease, myocardial infarction, congestive heart failure or coronary artery disease, angina, asthma, arthritis, stroke, and kidney disease), scored 1 point on the illness component. Kidney disease is excluded because of missing data in the NHIS. Due to insufficient data on weight change in both the Randomized Trial of Enteral Glutamine to Minimize the Effects of Burn Injury (RE-ENERGIZE) and National Health Interview Survey (NHIS) cohorts, loss of weight was replaced by malnutrition. Using accepted definitions of malnutrition from the World Health Organization and the European Society for Clinical Nutrition and Metabolism [21], individuals with a body mass index (BMI) lower than 18.5 kg/m^2^, those aged between 65–70 years with BMI < 20 kg/m^2^, and those aged over 70 years with BMI < 22 kg/m^2^ were assigned a score of 1 point on the malnutrition component. Finally, each component on the FRAIL scale contributes 1 point to overall scores ranging from 0–5, where a score of 0 indicates robustness, while scores between 1–2 indicate pre-frailty and scores between 3–5 indicate frailty

The FRAIL scale consists of 5 components: fatigue, resistance, ambulation, illness, and loss of weight [15]. This is a widely used and extensively studied tool with previous research supporting its validity in frailty assessment in various populations, although not specifically in a burn injury cohort [16–20]. On average, 3 to 6 months after hospital discharge, the patients were contacted to complete the 36-Item Short Form Health Survey (SF-36) questionnaire. Fatigue, resistance, and ambulation were determined from responses to the SF-36 or NHIS questionnaires and are indicative of the post-discharge status. Illness indicated the presence of more than 5 of the following conditions: hypertension, diabetes, cancer other than minor skin cancer, chronic lung disease, myocardial infarction, congestive heart failure or coronary artery disease, angina, asthma, arthritis, and stroke. Kidney disease was excluded from the analysis because of missing data in the NHIS. Due to insufficient data in both burn injury and control cohorts, the concept of “loss of weight” was substituted with “malnutrition”, using recognized definitions from the World Health Organization and the European Society for Clinical Nutrition and Metabolism [21]. Therefore, in terms of time point, the onset of illness and loss of weight occurred at the time of hospital admission, while fatigue, resistance, and ambulation were assessed at 3–6 months after hospital discharge. A score of 0 on the FRAIL scale indicates robustness, a score of 1–2 indicates pre-frailty, and a score of 3–5 indicates frailty. Figure 1 provides an overview of the questions and scoring methodology [21]. The relationship between TBSA and frailty was investigated by comparing the distribution of TBSA among three groups: robustness (*n* = 268), pre-frailty (*n* = 254), and frailty (*n* = 78).

### Outcomes

The post-discharge independence of burn patients was evaluated through the assessment of their responses to the Katz index of activities of daily living (ADL) [22] and the Lawton index of instrumental activities of daily living (iADL) [23] questionnaires, as previously described [19]. ADL and iADL are two commonly assessed domains on self-reported questionnaires for measuring functional disability [24], both considered significant predictors of long-term care service use [25]. ADL includes 4 components (toileting, transferring, continence, and feeding), while iADL comprises 8 components (ability to use a telephone, shopping, food preparation, housekeeping, laundry, mode of transportation, responsibility for own medications, and ability to handle finances). Any response indicating less than complete independence was classified as an ADL limitation. The utilization of both the Katz index for assessing ADL independence and the Lawton index for evaluating iADL has been prevalent in prior research due to its comprehensive insight into an individual’s functional abilities [26–31]. While, ADL to self-care tasks essential for basic survival and well-being, such as toileting, bathing, eating, and dressing, iADL involves more complex tasks supporting daily life within the home and community. Examples include tasks such as household management, financial administration, telephone usage, grocery procurement, and medication compliance [32]. The combined iADL and ADL scores were calculated and plotted against the follow-up period. For example, when computing the iADL score, each component was assigned a point score which was then summed to yield an overall score. For instance, the component “Ability to use the telephone” was scored as follows: “Operates telephone on own initiative, looks up and dials numbers” scored 0, “Dials a few well-known numbers” scored 1, “Answers telephone, but does not dial” scored 2, and “Does not use the phone at all” scored 3. Consequently, higher scores indicate greater dependence. ADL and iADL assessments were conducted 2 to 15 months post-hospital discharge.

### Statistical analysis

All data from both databases were collected and matched in Microsoft Excel^®^ 2024 (Microsoft, Redmond, WA, USA). Propensity score matching was performed in R software (version 4.1.2) using the Matchlt package. Each treated unit “burn patient” was paired with two controls “general population” through a nearest-neighbor one-to-two matching technique to enhance study precision as previously described [33]. Matching variables included age, sex, race/ethnicity, BMI, history of alcohol misuse, and current smoking status. The quality of the matching was visualized with histograms and jitter plots (Additional file 1: Figs. S1, S2). The resulting matched cohorts were subsequently utilized for assessing frailty by comparing all 5 components of the FRAIL scale. Continuous data (age, BMI) were presented as means and standard deviations (SD) and compared using a Student’s *t*-test, while categorical data were presented as absolute *n* (%) and compared using a *χ*^2^ or Fishers exact test, as appropriate. Finally, a multivariable linear regression analysis was performed on the burns cohort to identify factors associated with frailty, compromised ADL, and iADL. Included variables were age, alcohol misuse, smoking, type of burn (scald, chemical, other), BMI, TBSA, LOHS, glutamine administration, sex, and race. All statistical analysis was conducted in GraphPad Prism (version 9) and the data were visualized in GraphPad Prism and Adobe Illustrator. All *P*-values less than 0.05 were considered significant.

## Results

### Cohort demographics and characteristics

**Fig. 2:**
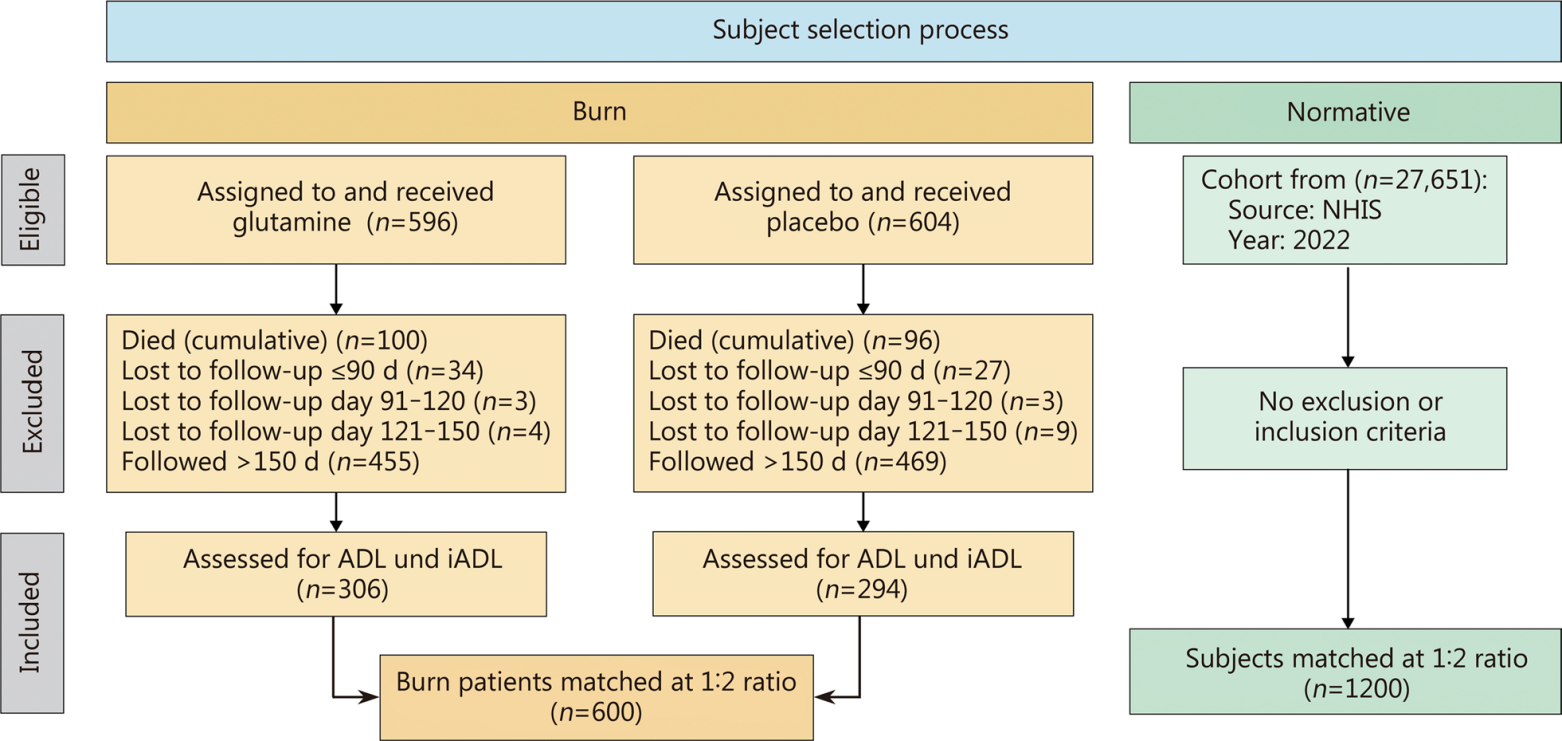
Patient recruitment process. ADL activities of daily living, iADL instrumental activities of daily living, NHIS National Health Interview Survey

In the RE-ENERGIZE trial, there were a total of 1200 participants, of whom 600 completed the necessary questionnaires to meet the eligibility criteria for this post-hoc study. The patient recruitment process is detailed in Fig. 2. Average follow-up time was (5.5 ± 2.3) months post-burn. A total of 1200 adults from the general population were included matched (Table 1). The burn population was well-matched to the general population, with both cohorts consisting predominantly of males (> 70.0%) and individuals of White ethnicity (> 70%). The cohorts were similar in terms of age [(48.7 ± 17.1) years vs. (48.3 ± 17.8) years, *P* = 0.65] and BMI [(28.3 ± 6.0) kg/m^2^ vs. (28.0 ± 7.6) kg/m^2^, *P* = 0.50]. The burn cohort had a higher percentage of Native American subjects (3.0% vs. 1.0%, *P* = 0.002), while the normative cohort had a higher percentage of Black or African American subjects (6.3% vs. 10.8%, *P* = 0.002; Table 1).

**Table 1:** Demographics and characteristics profiles of the groups after propensity score matching

Characteristic	Burn (*n* = 600)	Normative (*n* = 1200)	*P*-value
Sex [*n* (%)]			0.57
Male	441 (73.5)	866 (72.2)	
Female	159 (26.5)	334 (27.8)	
Age (years, mean ± SD)	48.7 ± 17.1	48.3 ± 17.8	0.65
BMI (kg/m^2^, mean ± SD)	28.3 ± 6.0	28.0 ± 7.6	0.50
Glutamine administration [*n* (%)]	294 (49.0)	NA	
TBSA (%, mean ± SD)	30.6 ± 15.1	NA	
LOHS (d, mean ± SD)	42.2 ± 24.9	NA	
Race [*n* (%)]			
Asian or Pacific Islander	32 (5.3)	70 (5.8)	0.67
Black or African American	38 (6.3)	129 (10.8)	0.002
Hispanic	50 (8.3)	127 (10.6)	0.13
Native American	18 (3.0)	12 (1.0)	0.002
White or Caucasian	456 (76.0)	844 (70.3)	0.01
Other	6 (1.0)	18 (1.5)	0.38
Other co-morbidities [*n* (%)]			
Smoking (current)	170 (28.3)	357 (29.8)	0.53
Alcohol abuse	67 (11.2)	138 (11.5)	0.83
Outcomes [*n* (%)]			
FRAIL score			
Robustness (0)	268 (44.7)	950 (79.2)	< 0.0001
Pre-frailty (1–2)	254 (42.3)	238 (19.8)	< 0.0001
Frailty (3–5)	78 (13.0)	12 (1.0)	< 0.0001
FRAIL scale components			
Fatigue	155 (25.8)	162 (13.5)	< 0.0001
Resistance	204 (34.0)	32 (2.7)	< 0.0001
Ambulation	251 (41.8)	46 (3.8)	< 0.0001
Illness	7 (1.2)	34 (2.8)	0.03
Loss of weight/malnutrition	14 (2.3)	31 (2.6)	0.75
FRAIL illnesses^1^			
Hypertension	150 (25.0)	377 (31.4)	0.005
Diabetes	72 (12.0)	94 (7.8)	0.004
Cancer other than minor skin cancer	99 (16.5)	125 (10.4)	0.0002
COPD/chronic lung disease	20 (3.3)	77 (6.4)	0.006
Myocardial infarction	14 (2.3)	51 (4.3)	0.04
CHF or CHD	13 (2.2)	68 (5.7)	0.0007
Angina	4 (0.7)	21 (1.8)	0.06
Asthma	26 (4.3)	162 (13.5)	< 0.0001
Arthritis	19 (3.2)	253 (21.1)	< 0.0001
Stroke	13 (2.2)	38 (3.2)	0.23

^1^Kidney disease was excluded from the illness analysis because of missing data in the NHIS. *BMI* body mass index, *TBSA* total body surface area, *LOHS* length of hospital stay, *CHF* congestive heart failure, *CHD* coronary heart disease, *COPD* chronic obstructive pulmonary disease, *NHIS* National Health Interview Survey

### Prevalence of frailty

Out of 600 burn patients, there were 268 classified as robustness, while 254 were categorized as pre-frailty, and another 78 as frailty individuals within this cohort group. Additionally, burn patients exhibited a notably lower likelihood of being classified as robustness compared to their counterparts in normative populations (44.7% vs. 79.2%, *P* < 0.0001), but showed a substantially higher probability of being categorized as pre-frail (42.3% vs. 19.8%, *P* < 0.0001), or frail individuals (13.0% vs. 1.0%, *P* < 0.0001; Table 1). Furthermore, when examining specific components of the FRAIL scale among these patients with burns, it became evident that they had an increased tendency towards experiencing fatigue (25.8% vs. 13.5%, *P* < 0.0001), increased resistance (34.0% vs. 2.7%, *P* < 0.0001), and restricted ambulation (41.8% vs. 3.8%, *P* < 0.0001). Moreover, the incidence of illness appeared higher in the general population compared to that observed among those with burns (1.2% vs. 2.8%, *P* = 0.03). However, malnutrition rates did not display significant differences between these two groups (2.3% vs. 2.6%, *P* = 0.75; Table 1). Lastly, an analysis focusing on various comorbidities encompassed within the illness component revealed that individuals from general populations demonstrated a notably greater likelihood of having conditions such as asthma, arthritis, congestive heart failure (CHF) or coronary heart disease (CHD), chronic obstructive pulmonary disease (COPD)/chronic lung disease, hypertension and myocardial infarction (*P* < 0.05) whereas those from the burn patient group displayed a markedly elevated probability for cancer other than minor skin cancer and diabetes (*P* < 0.01).

### TBSA and frailty in the burn population

**Fig. 3:**
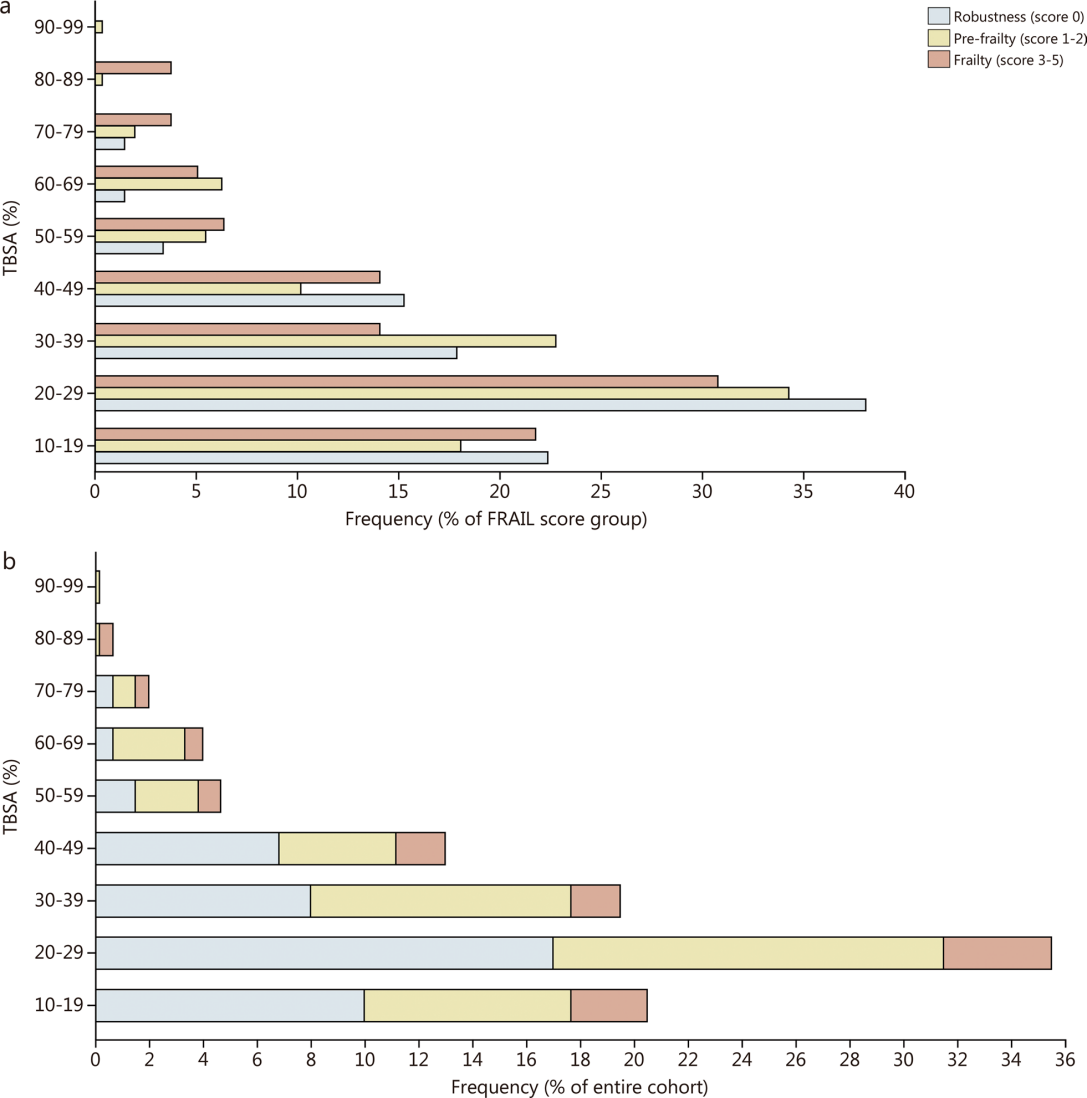
Association between TBSA and frailty. **a** The distribution of TBSA percentages across frailty score groups indicates that the majority of robust, pre-frail, and frail patients had a TBSA between 20 and 29%. As TBSA increases, there is a corresponding increase in the percentage of pre-frail and frail patients. **b** The distribution of TBSA among the patient cohort is visualized as a percentage. The majority of patients demonstrated robustness with a TBSA of 20–29% (102/600; 17.0% of all patients), followed by pre-frail patients within the same TBSA range (87/600; 14.5% of all patients)

When examining the relationship between TBSA and frailty, we observed that the majority of robust patients (score 0) had a TBSA ranging from 20 to 29% (102/268; accounting for 38.1% of all robust patients), followed by a TBSA range of 10 to 19% (60/268; representing 22.4% of all robust patients). The overall TBSA range for robust patients was from 10 to 76%. Similarly, most pre-frail patients (score 1–2) exhibited a TBSA between 20 and 29% (87/254; constituting 34.3% of all pre-frail patients), followed by a TBSA between 30 and 39% (58/254; representing 22.8% of all pre-frail patients). The total TBSA range for pre-frail patients was from 10 to 93%. Finally, the majority of frail patients (score 3–5) had a TBSA between 20 and 29% (24/78; accounting for 30.8% of all frail patients), followed by a TBSA ranging from 10 to 19% (17/78; constituting 21.8% of all frail patients). The total TBSA range for frail patients was from 10 to 85% (Fig. 3a). When considering the entire cohort, most patients exhibited robust and had a TBSA of 20–29% (102/600; 17.0% of all patients), followed by pre-frail patients with a TBSA of 20–29% (87/600; 14.5% of all patients), and then robust patients with a TBSA of 10–19% (60/600; 10.0% of all patients; Fig. 3b).

### ADL in the burn population

In comparison to robust burn patients, pre-frail burn patients exhibited a significantly higher likelihood of requiring assistance in toileting (11.4% vs. 2.6%, *P* < 0.0001), transferring (10.6% vs. 0.7%, *P* < 0.0001), continence (9.1% vs. 1.1%, *P* < 0.0001), and feeding (6.7% vs. 1.1%, *P* < 0.0001). Similarly, frail burn patients were notably more likely than robust patients to necessitate assistance in toileting (30.8% vs. 2.6%, *P* < 0.0001), transferring (28.2% vs. 0.7%, *P* < 0.0001), continence (20.5% vs. 1.1%, *P* < 0.0001), and feeding (17.9% vs. 1.1%, *P* = 0.001; Table 2). The distribution of ADL score over follow-up time is depicted in Additional file 1: Fig. S3.

**Table 2:** Burn patients’ responses to the questionnaires on activities of daily living (ADL) and instrumental activities of daily living (iADL) [*n* (%)]

Activity	Robustness (*n* = 268)	Pre-frailty (*n* = 254)	Frailty (*n* = 78)	^a^*P*-value	^b^*P*-value
ADL					
Toileting				< 0.0001	< 0.0001
Goes to the toilet, gets on/off, arranges clothes, cleans genital area without help	257 (95.9)	219 (86.2)	54 (69.2)		
Needs help transferring to the toilet, cleaning themself, or using bed pan/commode	7 (2.6)	29 (11.4)	24 (30.8)		
No response	4 (1.5)	6 (2.4)	0		
Transferring				< 0.0001	< 0.0001
Moves in/out of bed/chair unassisted. Mechanical aids are acceptable	262 (99.2)	221 (87.0)	56 (71.8)		
Needs help in moving from bed to chair or requires a complete transfer	2 (0.7)	27 (10.6)	22 (28.2)		
No response	4 (1.5)	6 (2.4)	0		
Continence				< 0.0001	< 0.0001
Exercises complete self-control over urination and defecation	261 (97.4)	225 (88.6)	62 (79.5)		
Is partially or incontinent of bowel or bladder	3 (1.1)	23 (9.1)	16 (20.5)		
No response	4 (1.5)	6 (2.4)	0		
Feeding				0.001	< 0.0001
Gets food into the mouth without help. Food prep may be done by another person	261 (97.4)	231 (90.9)	64 (82.1)		
Needs partial or total help with feeding or requires parenteral feeding	3 (1.1)	17 (6.7)	14 (17.9)		
No response	4 (1.5)	6 (2.4)	0		
iADL					
Ability to use the telephone				0.002	< 0.0001
Operates telephone on own initiative, looks up and dials numbers	256 (95.5)	221 (87.0)	63 (80.8)		
Dials a few well-known numbers	5 (1.9)	10 (3.9)	2 (2.6)		
Answers telephone, but does not dial	3 (1.1)	7 (2.8)	5 (6.4)		
Does not use the telephone at all	0	10 (3.9)	8 (10.3)		
No response	4 (1.5)	6 (2.4)	0		
Shopping				< 0.0001	< 0.0001
Takes care of all shopping needs independently	219 (81.7)	123 (48.4)	17 (21.8)		
Shops independently for small purchases	25 (9.3)	51 (20.1)	11 (14.1)		
Needs to be accompanied on any shopping trip	13 (4.9)	40 (15.7)	25 (32.1)		
Completely unable to shop	6 (2.2)	34 (13.4)	25 (32.1)		
No response	5 (1.9)	6 (2.4)	0		
Meal preparation				< 0.0001	< 0.0001
Plans, prepares, and serves adequate meals independently	218 (81.3)	137 (53.9)	21 (26.9)		
Prepares adequate meals if supplied with ingredients	24 (9.0)	33 (13.0)	16 (20.5)		
Heats prepared meals/prepare meals but diet not adequate	13 (4.9)	22 (8.7)	7 (9.0)		
Needs to have meals prepared and served	9 (3.4)	56 (22.0)	34 (43.6)		
No response	4 (1.5)	6 (2.4)	0		
Housekeeping				< 0.0001	< 0.0001
Maintains house alone with occasion assistance (heavy work)	194 (72.4)	94 (37.0)	11 (14.1)		
Performs light daily tasks such as dishwashing, bed making	47 (17.5)	72 (28.3)	19 (24.4)		
Performs light daily tasks; cannot maintain acceptable cleanliness	8 (3.0)	27 (10.6)	7 (9.0)		
Needs help with all home maintenance tasks	4 (1.5)	20 (7.9)	11 (14.1)		
Does not participate in any housekeeping tasks	11 (4.1)	35 (13.8)	30 (38.5)		
No response	4 (1.5)	6 (2.4)	0		
Laundry				< 0.0001	< 0.0001
Does personal laundry independently	218 (81.3)	154 (60.6)	26 (33.3)		
Launder small items, rinse socks/stockings	29 (10.8)	27 (10.6)	14 (17.9)		
All laundry must be done by others	17 (6.3)	66 (26.0)	38 (48.7)		
No response	4 (1.5)	7 (2.8)	0		
Travel				< 0.0001	< 0.0001
Travels independently on public transportation or drives own car	220 (82.1)	140 (55.1)	27 (34.6)		
Arrange travel via taxi, do not otherwise use public transportation	20 (7.5)	19 (7.5)	7 (9.0)		
Travels on public transportation when assisted/accompanied	5 (1.9)	20 (7.9)	3 (3.8)		
Travel limited to taxi or automobile with the assistance of another	16 (6.0)	51 (20.1)	26 (33.3)		
Does not travel at all	3 (1.1)	18 (7.1)	15 (19.2)		
No response	4 (1.5)	6 (2.4)	0		
Medication management				< 0.0001	< 0.0001
Is responsible for taking medication in correct dosages at the correct time	249 (92.9)	186 (73.2)	40 (51.3)		
Is responsible if medication prepared in advance in separate dosages	13 (4.9)	43 (16.9)	19 (24.4)		
Is not capable of dispensing own medication	2 (0.8)	19 (7.5)	19 (24.4)		
No response	4 (1.5)	6 (2.4)	0		
Ability to manage finances				< 0.0001	< 0.0001
Independent (budgets, pays rent/bills, goes to bank); collects/tracks income	237 (88.4)	176 (69.3)	38 (48.7)		
Manages day-to-day purchases; helps with banking/major purchases	21 (7.8)	40 (15.7)	21 (26.9)		
Incapable of handling money	5 (1.9)	32 (12.6)	19 (24.4)		
No response	5 (1.9)	6 (2.4)	0		

^a^*P*-value: pre-frailty vs. robustness; ^b^*P*-value: frailty vs. robustness

### iADL in the burn population

Compared to robust patients, pre-frail burn patients demonstrated significantly lower levels of independently in using the telephone (87.0% vs. 95.5%, *P* = 0.002), shopping (48.4% vs. 81.7%, *P* < 0.0001), meal preparation (53.9% vs. 81.3%, *P* < 0.0001), housekeeping (37.0% vs. 72.4%, *P* < 0.0001), laundry (60.6% vs. 81.3%, *P* < 0.0001), travel (55.1% vs. 82.1%, *P* < 0.0001), managing their medication (73.2% vs. 92.9%, *P* < 0.0001), and financial management (69.3% vs. 88.4%, *P* < 0.0001; Table 2).

In comparison to robust patients, frail burn patients showed significantly lower levels of independence in using the telephone (80.8% vs. 95.5%, *P* < 0.0001), shopping (21.8% vs. 81.7%, *P* < 0.0001), preparing meals (26.9% vs. 81.3%, *P* < 0.0001), housekeeping (14.1% vs. 72.4%, *P* < 0.0001), laundry (33.3% vs. 81.3%, *P* < 0.0001), travel (34.6% vs. 82.1%, *P* < 0.0001), managing their medication (51.3% vs. 92.9%, *P* < 0.0001), and financial management (48.7% vs. 88.4%, *P* < 0.0001; Table 2). The distribution of iADL score against follow-up time is shown in Additional file 1: Fig. S3.

### Factors associated with frailty and limitations in ADL or iADL in the burn cohort

The entire burn cohort was utilized to conduct a multivariable linear regression analysis aimed at identifying risk factors for frailty, as well as limitations in ADL and iADL. The results revealed that age (*P* < 0.0001) and smoking (*P* = 0.04) were independent risk factors for frailty. Furthermore, the chemical burn was identified as an independent risk factor for ADL limitations (*P* = 0.0003), while both chemical burn (*P* = 0.01) and scald burn (*P* = 0.04) were identified as independent risk factors for iADL limitations. Additionally, individuals of Asian or Pacific Islander race were found to have a protective effect against iADL limitations (*P* = 0.01, Table 3).

**Table 3:** Multivariate assessment of frail status, ADL and iADL challenges

Primary outcomes	Estimate	95%CI	*P*-value
Frail status			
Age	0.01	0.01–0.02	< 0.0001
Alcohol abuse	0.21	−0.12 to 0.54	0.21
Smoking	0.23	0.01–0.45	0.04
Burn (scald)	−0.04	−0.42 to 0.34	0.83
Burn (chemical)	−0.13	−0.85 to 0.58	0.71
Burn (other)	−0.43	−1.64 to 0.79	0.49
BMI	0.01	0.00–0.03	0.15
TBSA	0.01	0.00–0.02	0.13
LOHS	0.00	0.00–0.01	0.22
Glutamine administration	0.02	−0.18 to 0.23	0.82
Sex (female)	0.18	−0.06 to 0.41	0.14
Race (native)	0.56	−0.07 to 1.20	0.08
Race (Black or African American)	0.34	−0.09 to 0.77	0.12
Race (Asian or Pacific Islander)	0.31	−0.11 to 0.74	0.15
Race (Hispanic)	0.01	−0.35 to 0.37	0.95
Race (other)	0.70	−0.25 to 1.65	0.15
ADL difficulties			
Age	0.00	0.00–0.00	0.98
Alcohol abuse	0.06	−0.05 to 0.16	0.32
Smoking	−0.02	−0.09 to 0.05	0.60
Burn (scald)	0.00	−0.12 to 0.13	0.96
Burn (chemical)	0.44	0.20–0.67	0.0003
Burn (other)	0.22	−0.18 to 0.62	0.29
BMI	0.00	−0.01 to 0.01	0.88
TBSA	0.00	0.00–0.01	0.18
LOHS	0.00	0.00–0.01	0.19
Glutamine administration	−0.03	−0.10 to 0.03	0.35
Sex (female)	0.01	−0.07 to 0.09	0.79
Race (native)	0.00	−0.21 to 0.21	0.98
Race (Black or African American)	0.05	−0.09 to 0.19	0.49
Race (Asian or Pacific Islander)	0.00	−0.14 to 0.14	0.96
Race (Hispanic)	0.06	−0.06 to 0.18	0.30
Race (other)	0.07	−0.24 to 0.39	0.64
iADL difficulties			
Age	0.00	0.35–0.98	0.67
Alcohol abuse	0.01	0.00–0.00	0.94
Smoking	0.04	−0.15 to 0.16	0.48
Burn (scald)	0.19	−0.07 to 0.14	0.04
Burn (chemical)	0.42	0.01–0.37	0.01
Burn (other)	0.12	0.09–0.75	0.69
BMI	0.00	−0.45 to 0.68	0.95
TBSA	0.00	−0.01 to 0.01	0.52
LOHS	0.00	−0.01 to 0.00	0.49
Glutamine administration	−0.07	0.00–0.00	0.13
Sex (female)	−0.01	−0.17 to 0.02	0.88
Race (native)	−0.12	−0.12 to 0.10	0.43
Race (Black or African American)	−0.08	−0.42 to 0.18	0.41
Race (Asian or Pacific Islander)	−0.25	−0.29 to 0.12	0.01
Race (Hispanic)	0.04	−0.45 to −0.05	0.68
Race (other)	−0.33	−0.13 to 0.20	0.14

*ADL* activities of daily living, *iADL* instrumental activities of daily living, *BMI* body mass index, *TBSA* total body surface area, *LOHS* length of hospital stay

## Discussion

Advancements in both burn care and intensive care have led to decreased mortality rates, with reports showing a survival rate of 96.7% among individuals treated at burn centers across the U.S. [1]. There has been a notable increase in post-burn morbidity within the expanding community of burn survivors [34, 35]. Put differently, as these survivors live longer lives, they are increasingly confronted with enduring consequences from their injuries. Frailty, characterized by diminished physiological reserve and increased susceptibility to stressors, can significantly complicate efforts to manage and rehabilitate these individuals (Additional file 1: Fig. S4) [11, 36]. This underscores the necessity for an enhanced comprehension regarding long-term susceptibility to frailty in this patient population. In the discussion, we leverage findings from this study to propose diverse strategies aimed at mitigating and limiting frailty among burn survivors.

### Early recognition and assessment

**Fig. 4:**
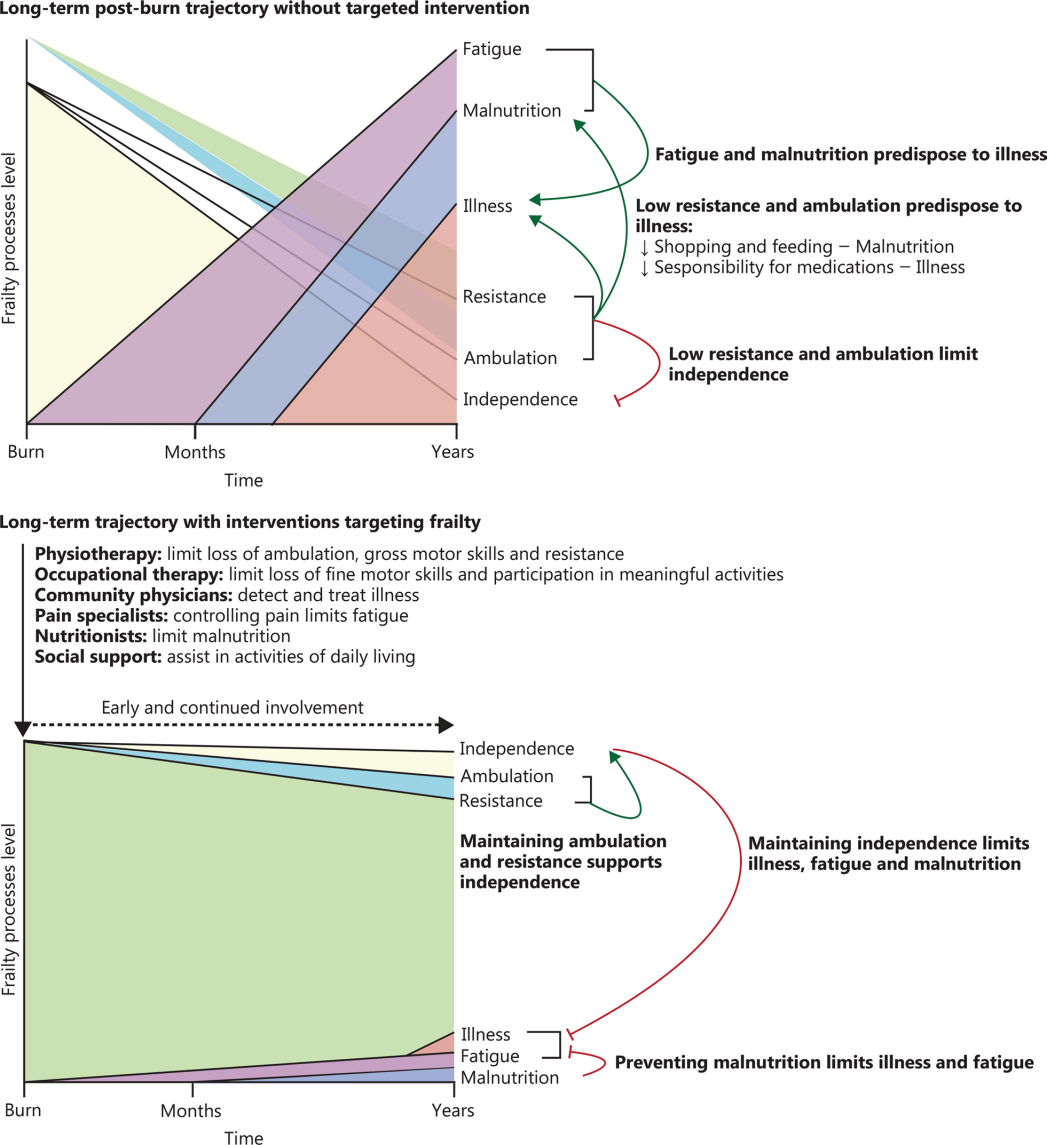
Long-term trajectory of frailty among burn patients is influenced by targeted intervention. Following burn injuries in the short term, patients may face compromised resistance and ambulation while concurrently experiencing fatigue stemming from various factors such as inadequate pain management leading to disrupted sleep patterns or heightened anxiety and depression related to trauma-induced sequelae. Collectively impacting patient independence including (instrumental) activities of daily living, these challenges encompass essential tasks, such as shopping and cooking, cleaning and managing medications, as well as finances. In the long run, this could result in malnutrition, an increase in chronic illnesses, and a notable elevation across all 5 components measured by the FRAIL score. Hypothetically, prompt long-standing engagement with a multidisciplinary team comprising rehabilitation services, community physicians, nutritionists, pain and mental health specialists, as well as social support, holds promise for mitigating fatigue, resistance compromises, and averting prolonged illness and malnutrition

Our analysis indicates a higher prevalence of frailty in the burn population, approximately 5 months after injury (Fig. 4). The components of fatigue, resistance, and ambulation were all significantly more restricted in the burn survivors, while the variables of frailty assessed on hospital admission, that is, illness and malnutrition, were higher or did not differ in the normative population, respectively. This provides evidence that although the patients were not frail upon admission to the hospital, they experienced a significant increase in frailty shortly after discharge. This emphasizes the importance of early recognition and assessment of frailty following a burn injury as a critical component of comprehensive burn care. Importantly, the RE-ENERGIZE data did not include information on the pre-burn frail status of patients. Therefore, an exact inference about which patients became frail after the injury cannot be made.

Interestingly, our multivariable analysis of predisposing factors did not reveal TBSA to have a significant impact on the development of frailty, while age was a predictable predictor. Although there was a slight trend suggesting that patients with higher TBSA were also more likely to have higher FRAIL scores (Fig. 3), the multivariate analysis showed no significant correlation between TBSA and frailty. TBSA is one of the most powerful indicators of burn trauma severity and strongly correlates with adverse outcomes and short- and long-term morbidity [37, 38]. This finding highlights the multifactorial nature of frailty in general and certainly emphasizes the need for more advanced screening methods that go beyond mere burn size [39–41].

### Multidisciplinary approach

The management of severe burn injuries in general, and frailty in severely burned patients in particular, requires a multidisciplinary team approach involving burn specialists, community physicians, geriatricians, pain management experts, physiotherapists, occupational therapists, mental health professionals, nutritionists, and social workers (Fig. 4).

The long-term trajectory of burn survivors is characterized by low resistance and low ambulation which limits patients’ independence, predisposing to illness. At the same time increased fatigue and malnutrition also predispose to illness. A multidisciplinary collaborative effort ensures comprehensive care for addressing the diverse needs of frail patients, including rehabilitation to minimize the loss of ambulation and resistance, medical management to promptly identify and treat illnesses such as cardiovascular compromise and diabetes, nutritional support to prevent malnutrition and thus limit illness, and psychosocial interventions [42]. By maintaining ambulation and resistance, independence is maximized which in turn limits malnutrition, illness and fatigue. For example, occupational therapy can promote well-being through occupation by enabling burn survivors to engage in meaningful activities of everyday life. Occupational therapy achieves this by collaborating with patients and their community to enhance the survivors’ ability to engage in their chosen or necessary occupation or by modifying their occupation or environment to better support their engagement [43].

We have identified significant limitations in iADL and ADL among frail burn survivors, which compromise their independence and predispose these patients to further frailty. Approximately 25% of frail burn survivors report being unable to dispense their medication, compared to 1% of robust survivors. This notable limitation may have multiple contributing factors (e.g., disability due to a hand burn, presence of compression garments, and the type and shape of medication bottles), presenting a multidisciplinary challenge for resolution. The inability to adhere to medications could result in untreated illness and consequently increase frailty [44, 45]. Addressing this issue may require modified medication bottles, adaptive aids promoting independence, as well as specialized situational training through physiotherapy and occupational therapy. Additionally, social support can enhance adherence and overall well-being. The retrospective nature of this post hoc analysis limited our ability to consider certain variables that may influence our results, such as the body location of the burn injury (e.g., extremities or face), depth of the burn (e.g., second or third degree), setting of the injury (e.g., work-related or home-related), post-discharge care options (e.g., inpatient physiotherapy, outpatient physiotherapy or occupational therapy, treatment by mental health or pain specialists), as well as the socioeconomic status of the patients.

### Optimizing nutrition and rehabilitation

Frailty often leads to impaired nutritional status and decreased muscle mass [46, 47], which can impede wound healing and functional recovery in burn patients [48, 49]. Our multivariable analysis revealed no association between glutamine administration and the extent of frailty, ADL, and iADL. Similarly, previous literature on amino acid supplementation in the treatment of sarcopenia and frailty has yielded conflicting results [50–52]. Although hydroxyl-methyl butyrate has been reported to enhance muscle protein synthesis when combined with arginine and glutamine, glutamine alone has not been shown to prevent muscle deterioration [53]. Following a burn injury, the body’s energy and nutrient demand increase dramatically to support tissue repair and wound healing [5]. During the acute phase, adequate nutrition, including sufficient calories, protein, vitamins, and minerals, is crucial for facilitating the regeneration of damaged tissues, while minimizing complications such as infections, and promoting faster wound closure [54]. Preventing malnutrition during this high-demand period can reduce the risk of long-term frailty associated with prolonged healing and impaired tissue integrity [55]. In the medium- and long-term, severe burn injuries result in muscle wasting and loss of lean body mass due to increased protein breakdown and decreased protein synthesis [56].

In turn, a significant reduction in muscle mass and strength can severely impact the activity and energy levels of burn survivors. They often experience chronic fatigue, decreased stamina, and overall weakness, making it challenging to perform even basic ADLs such as dressing and bathing [57]. Our findings indicated that frailer burn survivors are more likely to require assistance with feeding, rely on parenteral feeding, and need help with shopping. More than 40% of frail burn survivors required their meals to be prepared and served compared to only 3% of robust survivors. Furthermore, diminished energy and physical capacity compromise the ability to participate in social and recreational activities, further affecting the quality of life [58]. As a result, survivors may increasingly depend on caregivers or adaptive aids, impacting their sense of independence and self-esteem. Additionally, the combination of physical limitations and increased dependency can contribute to a cycle of reduced physical activity, further exacerbating muscle wasting and frailty. Optimal nutrition, particularly high-quality protein intake, is crucial for preserving muscle mass, strength, and function, which are essential for mobility, independence, and overall resilience against frailty (Additional file 1: Fig. S4) [59]. Adequate nutrition is essential for providing the energy required for daily activities, rehabilitation, and physical therapy. It plays a crucial role in maintaining muscle strength, endurance, and functional independence. These factors are fundamental prerequisites for implementing a comprehensive rehabilitation program that includes nutritional support, physical therapy, and occupational therapy to help mitigate these effects [58]. Such programs aim to restore muscle mass, improve energy levels, and enhance the individual’s ability to participate in ADL, ultimately promoting greater independence and quality of life.

### Long-term follow-up and care

Burn patients are at increased risk of experiencing long-term complications, such as chronic pain, functional impairment, and recurrent hospitalizations, which have been shown to contribute to frailty (Additional file 1: Fig. S4) [8, 35, 39]. In a recent post hoc analysis of the same burn cohort, our research team identified a strong correlation between chronic pain, anxiety, and depression in these patients [60]. Previous research has also established a significant link between poor mental health and long-term frailty [61]. This highlights the necessity for comprehensive care that integrates both mental health support and physical rehabilitation to optimize the long-term outcomes of burn survivors. Establishing structured long-term follow-up programs is essential for monitoring progress, addressing ongoing needs, and preventing future frailty-related events. Losing track of burn patients during follow-up poses a significant concern both clinically and scientifically: comparison of the burn patients who were “lost to follow-up” with those included as responders revealed notable differences in baseline characteristics. Non-responders were on average younger, more likely to be smokers, less likely to be Hispanic but more likely to be African American. They were also more likely to have had inhalation injury or been transferred to another hospital ward with shorter hospital and ICU stays. This inherent limitation present in all prospective research regarding short- and long-term burn survivors emphasizes the importance of standardized 6-month follow-ups that extend beyond evaluating the need for secondary reconstructive procedures.

Although early detection of frailty and ADL deficiencies has been established, little information exists regarding their longitudinal progression. It is essential to collect outcome measures over time [62, 63], and implement extended, standardized, and interdisciplinary long-term protocols. A recent meta-analysis found that follow-up for burn patients seldom extends 5 years post-injury, which is considered long term [39]. Currently, all specialized follow-up care for burn survivors is exclusively provided by surgical institutions involved in secondary reconstruction. After achieving satisfactory reconstruction and reaching an acceptable level of scarring control, pain management effectiveness, and functional capability improvement, patient monitoring becomes sporadic or ceases altogether. There remains a lack of comprehensive protocols for diagnosing and treating long-term organ damage across disciplines while assessing their respective contributions to complex phenomena such as frailty. It is essential to identify long-term complications that may appear unrelated but are linked with an increased risk of developing diabetes and cardiovascular disease over decades. The establishment of a standardized interdisciplinary system for monitoring burn patients in the long term is crucial. This system could involve lifelong, follow-up appointments every 5 years similar to those offered for cancer patients after hospital discharge. To validate our preliminary findings comprehensively and ensure their applicability across different contexts, independent studies or datasets should confirm them. Conducting a prospective study comparing frailty levels upon admission with those at early (within months) and long-term (after years) post-discharge intervals, while examining diverse post-discharge care options like physiotherapy or occupational therapy would significantly enhance our understanding of chronic burn injury effects.

### Limitations

The response data are based on self-reporting, which is inherently susceptible to inaccuracies. Due to differences in the questionnaire protocols of the two databases (RE-ENERGIZE and NHIS), certain questions and response options were adjusted to achieve consensus. Although the RE-ENERGIZE data spans 6 years (2016–2021), we chose to utilize only the 2022 NHIS data as it provided all the necessary frailty assessment information due to its rotating design. The use of a general population database and propensity score matching helps to minimize this limitation. Another limitation of our study is that while the NHIS data is US-centric, the RE-ENERGIZE data collection was international, with the majority of patients based in North America. Given that only a limited subset of both populations was sampled, generalizability poses a concern. The extent to which our results can be applied to other populations and settings, particularly low- and middle-income countries, remains uncertain. Baseline differences in co-morbidities were observed between the two groups. The existing literature on illness present on admission following an acute burn is limited and inconclusive [64, 65]. Therefore, further research is necessary to determine whether there are genuine disparities in the baseline health of acute burn injury patients compared to the general population. The specific body location is particularly relevant, as injuries to the head and neck, as well as upper and lower extremities, are associated with higher levels of disability and would consequently be linked with elevated FRAIL scores [66]. Although data on the exact depth of the burns were not provided, all eligible patients for the trial had partial- or full-thickness burns requiring surgery. Generalizability may also pose a concern since only a restricted subset of both populations was sampled. The burn cohort analyzed in this study was defined post hoc and had not been considered in the original power calculation of the sample size. Therefore, it has been previously proposed that statistical hypotheses from post hoc analyses are inherently regarded as exploratory only [67]. Finally, due to the cross-sectional design of the study, making causal inferences is precluded.

## Conclusions

In this study, we conducted an analysis of one of the largest multicenter cohorts of patients with extensive burns to determine the prevalence of frailty in such patients’ months after injury, comparing it to a non-burned general population. Patients with a history of burns exhibit a higher prevalence of frailty compared to the general population group, and these differences are apparent a few months post-discharge, which is typically when burn survivors return to their normal lives. Additionally, we investigated the interrelationship between the presence of frailty and compromise in (instrumental) ADL. By establishing the severity of the issue and describing its impact on quality of life, we aim to identify potential opportunities and avenues for guiding clinical practice, future research, and policymaking efforts.

## Supplementary Information

**Additional file 1: Fig. S1** Quality of matching visualized as a histogram. **Fig. S2** Quality of matching visualized as a jitter plot. **Fig. S3** ADL and iADL scores assessed over the follow-up period in months. **Fig. S4** Theoretic schematic depicting the potential impact of frailty on patients with a history of burn injury.

## Data Availability

Datasets generated and analyzed to provide the findings in this study are available from the corresponding author upon reasonable request.

## Abbreviations

ADL: Activities of daily living
BMI: Body mass index
CHF/CHD: Congestive heart failure/coronary heart disease
COPD: Chronic obstructive pulmonary disease
FRAIL: Fatigue, Resistance, Ambulation, Illness, Loss of weight
iADL: Instrumental activities of daily living
ICU: Intensive care unit
LOHS: Length of hospital stay
NHIS: National Health Interview Survey
RE-ENERGIZE: Randomized Trial of Enteral Glutamine to Minimize the Effects of Burn Injury
SF-36: 36-Item Short Form Health Survey questionnaire
TBSA: Total body surface area
