# Supplementary material for: Frailty as a sequela of burn injury: a post hoc analysis of the “RE-ENERGIZE” multicenter randomized-controlled trial and the National Health Interview Survey
Source: Mil Med Res. 2024 Sep 12;11:63. doi: 10.1186/s40779-024-00568-x (PMC11391741; doi:10.1186/s40779-024-00568-x)
Supplement: Supplementary file 1 — Additional file 1: Fig. S1 Quality of matching visualized as a histogram. Fig. S2 Quality of matching visualized as a jitter plot. Fig. S3 ADL and iADL scores assessed over the follow-up period in months. Fig. S4 Theoretic schematic depicting the potential impact of frailty on patients with a history of burn injury. [file 40779_2024_568_MOESM1_ESM.pdf]

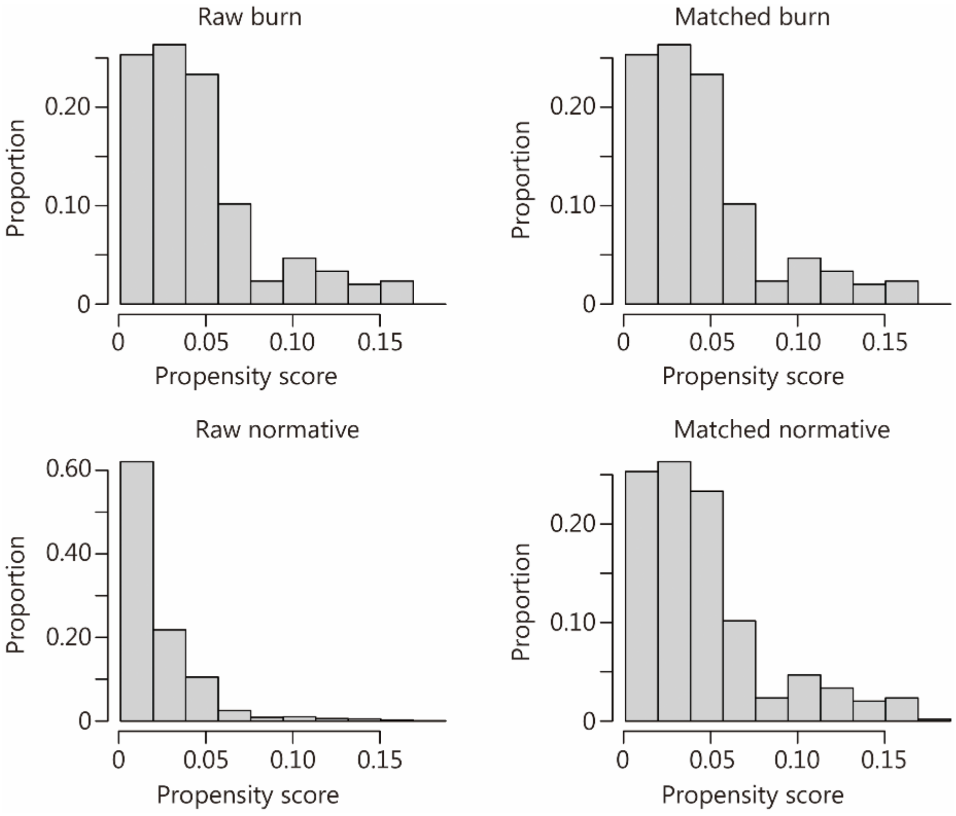

Fig. S1 Quality of matching visualized as a histogram. It shows the density of propensity score distribution in the burn and normative groups before and after matching. A comparison of the raw burn and raw normative histograms before propensity score matching reveals an unbalanced distribution. Following matching, the matched burn and normative groups display balanced histograms

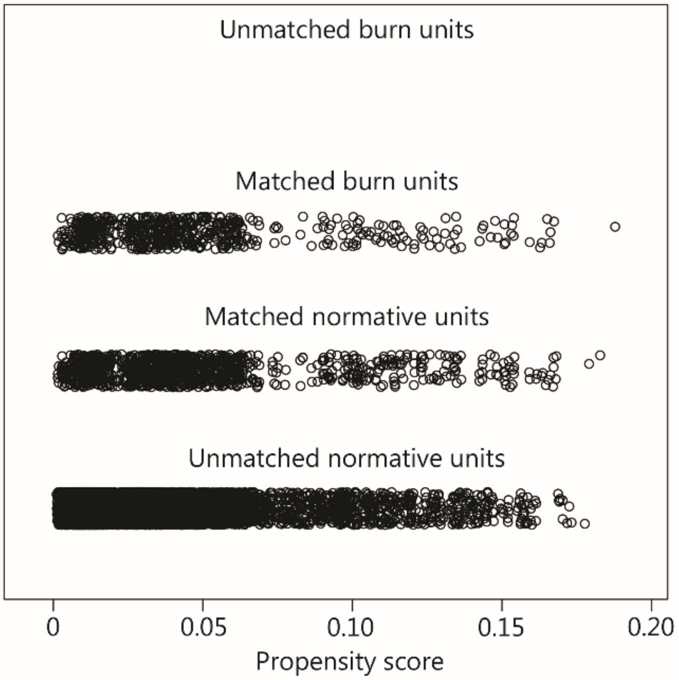

Fig. S2 Quality of matching visualized as a jitter plot. It displays the distribution of matched and unmatched data points in terms of propensity score values. Since this was a 1:2 (burn:normative) matching, and all burn points were successfully matched, there are no unmatched burn unit points. Low propensity scores in the normative group have been excluded

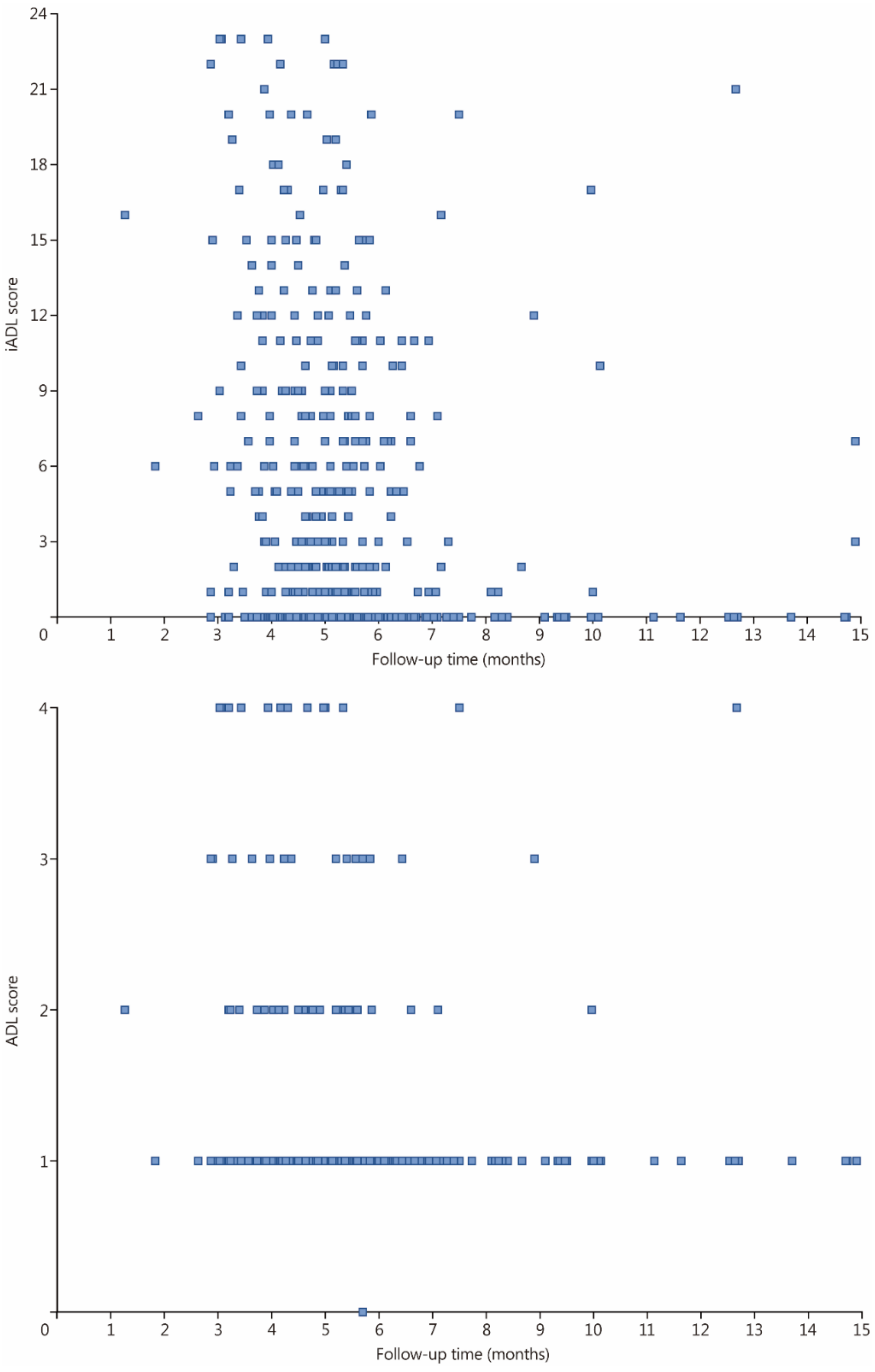

Fig. S3 ADL and iADL scores assessed over the follow-up period in months. Higher scores indicate greater dependence. involved assigning a score to each component, which was then totaled to yield an overall score. For instance, when calculating iADL, each component received a specific score. The “Ability to use the telephone” was scored based on the following criteria: 0 for “Operates telephone on own initiative, looks up and dials numbers”, 1 for “Dials a few well-known numbers”; 2 for “Answers telephone, but does not dial”; and 3 for “Does not use the telephone at all”

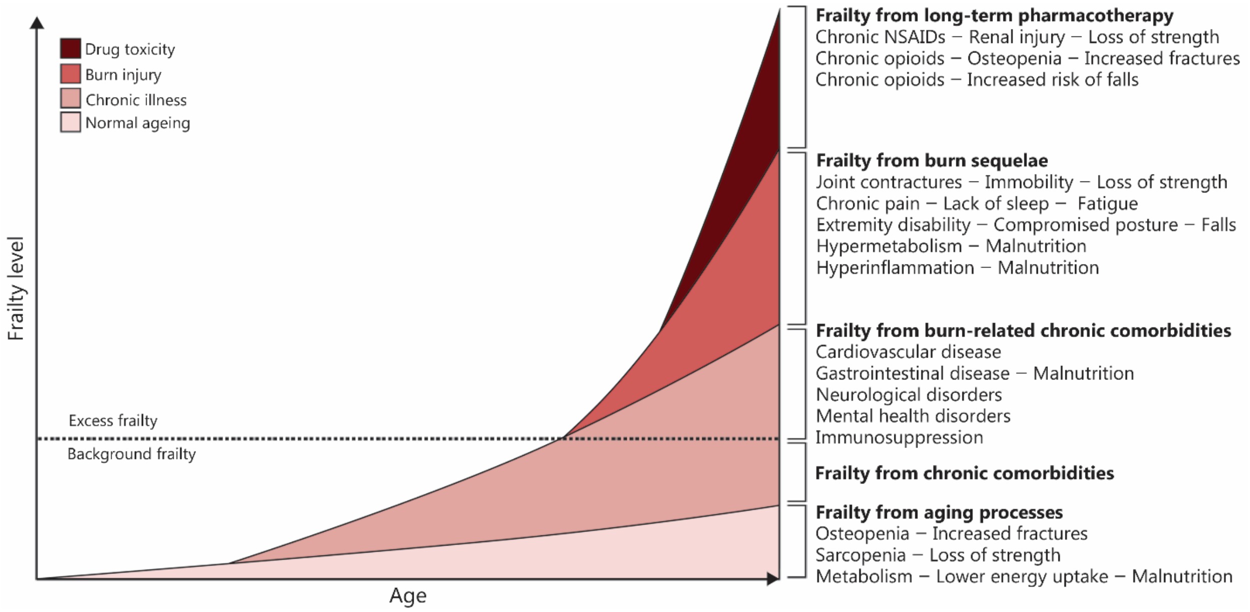

Fig. S4 Theoretic schematic depicting the potential impact of frailty on patients with a history of burn injury. As patients age, they typically experience a baseline level of frailty stemming from cellular senescence, inflammation, and chronic comorbidities, such as diabetes and heart disease. However, individuals with a history of burn injury may theoretically experience excess frailty due to 3 components: additional burn-related chronic illnesses (e.g., compromised cardiovascular comorbidities), direct sequelae of the burn injury (e.g., limited mobility), and the effects of long-term pharmacotherapy (e.g., potential toxicity from various pain medications). Modified from a figure by Goede et al. [36] illustrating the components of frailty in elder patients with hematological malignancies. NSAIDs non-steroidal anti-inflammatory drugs
